# Optimization of Chemical Bonding through Defect Formation and Ordering—The Case of Mg_7_Pt_4_Ge_4_

**DOI:** 10.1021/acs.inorgchem.2c04312

**Published:** 2023-05-19

**Authors:** Siméon Ponou, Sven Lidin, Anja-Verena Mudring

**Affiliations:** † Department of Materials and Environmental Chemistry, Stockholm University, Svante Arrhenius väg 16C, Stockholm 114 18, Sweden; ‡ Centre for Analysis and Synthesis, Department of Chemistry, Lund University, Naturvetarvägen 14, Box 124, Lund SE-22100, Sweden; § Intelligent Advanced Materials Group, Department of Biological and Chemical Engineering and iNANO, Aarhus University, Åbogade 40, Aarhus N 8200, Denmark

## Abstract

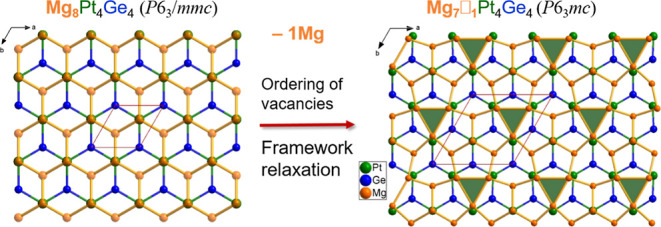

The new phase Mg_7_Pt_4_Ge_4_ (≡Mg_8_□_1_Pt_4_Ge_4_; □ = vacancy) was prepared by reacting a mixture of the corresponding elements at high temperatures. According to single crystal X-ray diffraction data, it adopts a defect variant of the lighter analogue Mg_2_PtSi (≡Mg_8_Pt_4_Si_4_), reported in the Li_2_CuAs structure. An ordering of the Mg vacancies results in a stoichiometric phase, Mg_7_Pt_4_Ge_4_. However, the high content of Mg vacancies results in a violation of the 18-valence electron rule, which appears to hold for Mg_2_PtSi. First principle density functional theory calculations on a hypothetical, vacancy-free “Mg_2_PtGe” reveal potential electronic instabilities at *E*_F_ in the band structure and significant occupancy of states with an antibonding character resulting from unfavorable Pt–Ge interactions. These antibonding interactions can be eliminated through introduction of Mg defects, which reduce the valence electron count, leaving the antibonding states empty. Mg itself does not participate in these interactions. Instead, the Mg contribution to the overall bonding comes from electron back-donation from the (Pt, Ge) anionic network to Mg cations. These findings may help to understand how the interplay of structural and electronic factors leads to the “hydrogen pump effect” observed in the closely related Mg_3_Pt, for which the electronic band structure shows a significant amount of unoccupied bonding states, indicating an electron deficient system.

## Abstract 2

Electronic structure-driven vacancy ordering in Mg_2−*x*_PtGe (*x* = 1/4) generates a 2 × 2 × 1 superstructure and triggers a relaxation of the defective 3D diamond-type Mg framework.

## Introduction

Intense research activities on polar intermetallic compounds (PICs) are motivated by their rich structural chemistry and outstanding physical properties, like superconductivity, thermoelectricity, and magnetocaloric effects.^1−8^ Some materials belonging to this class have become vital for innovative technologies such as renewable energy generation and storage or catalysis.^9−12^

The designation “polar” refers to the difference in electronegativity of the constituting elements.^1,2^ Electronically, PICs are located between Hume-Rothery and Zintl phases, often with *e*/*a* (valence electron count per atom) values around two.^1^ While, for valence electron poor Hume-Rothery (with *e*/*a* less than two) and electron precise Zintl phases, logical rules for electron count, formation, and, consequently, classification exist, the picture for PICs is still unclear. However, to a certain extent, structural and bonding features commonly observed for both nonpolar and Zintl phases are present in PICs.^2^ Often, a formal electron transfer according to their electronegativity differences from the “active” metal component to the more electronegative can be assumed. However, the metallic nature of these compounds indicates that significant electron back-donation from the electronegative to the electropositive component must take place.^13−17^

Of particular interest are intermetallics involving noble metals such as gold and platinum.^8,18^ These noble metals possess a high electronegativity, at par with those of heavier halogens and chalcogens.^19^ When combined with alkali metals, even salts such as CsAu (including solvates)^20,21^ and Cs_2_Pt^22^ as well as double salts such as Cs_7_Au_5_O_2_≡4CsAu·Cs_3_AuO_2_^23,24^ and Cs_9_Pt_4_H≡4Cs_2_Pt·CsH^25^ form. The high electronegativity originates from strong relativistic influences.^26^ Because of relativistic effects, the 6s orbitals are lowered in energy and the 5d are elevated, which gives these elements unique possibilities for the formation of structural motives, through both ionic and covalent bonding. Because of their peculiar bonding capabilities originating from relativity, noble metals like and their alloys are also of interest for catalysis, particularly for hydrogenation reactions.^27−31^

On the other hand, hydrogen storage materials are garnering interest for a safe hydrogen economy. Magnesium (Mg) is one of the most promising candidates among the diverse solid hydrogen storage materials due to its high gravimetric hydrogen capacity combined with very high abundance, non-toxicity, and low cost. However, the stable hydrogen carrier MgH_2_ can only desorb hydrogen at high temperatures (above 300 °C) due to its high formation enthalpy, and the hydrogen desorption kinetics is also sluggish. To address these challenges, the “hydrogen pump” effect is considered as one attractive method for improving the hydrogen desorption of MgH_2_.^32^ In this context, PIC Mg_3_Pt was recently identified in the Mg-based core–shell Mg@Pt nano-composite via in situ TEM (transmission electron microscopy), showing a remarkable “hydrogen pump” effect, as it can solubilize H atoms and transfer them, expediting the desorption rate of MgH_2_. However, very little is known regarding the structural or electronic factors behind the extraordinary properties of Mg_3_Pt.^32,33^ In this context, the relativistic effects on the chemical bonding are of interest^34−42^ and seem to be important for the excellent performance of Pt alloys as catalysts and hydrogen storage materials, and it is important to gain a deeper knowledge on the electronic structure of such materials. Interestingly, Mg_2_PtSi (Na_3_As-type, or more precisely the related ternary Li_2_CuAs type)^42^ is structurally very close to Mg_3_Pt (Cu_3_P type), and the former can be derived from the latter by the substitution of one Mg site by a Si atom.

In the course of our research efforts to investigate the bonding peculiarities of binary and ternary intermetallic phases with noble metals like Pt, we became interested in understanding the driving forces behind the structural dynamic in the series Mg_2_PtX (with X = Mg, Si). We started by exploring the hypothetical Ge analogue “Mg_2_PtGe” to verify its stability and structural peculiarities as compared to already reported Mg_2_PtSi and Mg_3_Pt.

Herein, we report on Mg_2–*x*_PtGe as the first structurally characterized phase in the Mg–Pt–Ge system. An unexpectedly large concentration of Mg defect in the system with a complete ordering for *x* = 0.25 is observed, yielding the stoichiometric phase Mg_7_Pt_4_Ge_4_ (space group *P*6_3_*mc*). Remarkably, like binary Mg_3_Pt, it also crystalizes in a 2 × 2 × 1 supercell with respect to the stoichiometric Si analogue Mg_2_PtSi but with a different symmetry. Theoretical band structure density functional theory calculations, using the linear muffin-tin-orbital (LMTO) code,^54,55^ help in identifying the structure stabilizing factors and bonding characteristics, in particular, the driving forces behind the unexpected formation of Mg vacancies and the violation of the usual 18 valence electron count.

## Experimental Section

### Synthesis and Analysis

The starting materials for the synthesis were the elements Mg (block, 99.999%; Alfa Aesar), Ge (pieces, 99.999%; American Elements), and Pt (pieces, 99.99%; from the Ames Laboratory), stored in an argon-filled glovebox and used as received. The mixtures (ca. 400 mg) with the atomic ratio Mg/Pt/Ge = 2:1:1 (by analogy to Mg_2_PtSi) were loaded on Ta ampoules (30 mm length and *Ø*: 6 mm) under an Ar atmosphere, sealed on both ends by arc melting. Variable amounts of Mg excess were added to compensate the evaporation under high temperatures and to control the Mg defect in the compound. The arc-sealed Ta ampoules were enclosed in a fused silica glass Schlenk tube under vacuum (ca. 10^–2^ mbar) to protect the ampoules from air oxidation at high temperatures. The reactions were carried out inside a programmable tube furnace by heating from room temperature up to 1000 °C over 10 h; after 1 h, the furnace was cooled slowly (2 °C/min) to 800 °C and the sample was annealed for five days. Finally, the furnace was cooled (6 °C/min) to room temperature. The reaction vessels were opened in air, revealing dark crystals with a trigonal prism shape and shiny metallic luster. The crystals were air and moisture stable and remained suitable for X-ray diffraction experiment after a couple of months. Routine phase analysis by powder X-ray diffraction on a Stoe Stadi MP diffractometer in the transmission mode [Ge(111) monochromator for Cu Kα_1_ radiation: λ = 1.54056 Å] equipped with a Mythen detector (linear position sensitive, PSD) confirmed the purity of the product with a nominal composition “Mg_2_PtGe”. The program suite WinXPow was employed for diffractometer control as well as data analysis.^43^

The chemical composition of single crystals of the title compound was verified by scanning electron microscopy (SEM) using a field emission scanning electron microscope (JSM-7000F, JEOL, Japan) operating at 15 kV and equipped with an energy dispersive X-ray spectrometer EDX system (INCAx-sight, Oxford Instruments, UK). The analysis of several single crystals of the title phases confirmed the presence of all three elements with the atomic ratios roughly consistent with the refined values.

### Single-Crystal X-ray Data Collection and Structural Refinement

For Mg_2–*x*_PtGe (*x* = 0.12), X-ray data were collected at room temperature on a Bruker SMART CCD diffractometer. The reflection intensities were integrated with the SAINT program in the SMART software package.^44^ Empirical absorption corrections were accomplished with the aid of the SADABS program.^45^ For Mg_2–*x*_PtGe (*x* = 0.25), i.e., Mg_7_Pt_4_Ge_4_, X-ray data were collected at an ambient temperature on a Xcalibur3 diffractometer with a CCD detector (Oxford Diffraction Ltd., UK), using graphite monochromatized Mo Kα radiation (λ = 0.71073 Å), operated at 50 kV and 40 mA, and a detector-to-crystal distance of 50 mm. A full set of data was obtained by ω-scan with 0.75° rotation width and 5 s exposure time per frame. Absorption correction based on a semi-empirical “multi-scan” approach was applied to the integrated reflections using the program CrysAlisPro from Agilent Technologies.^46^ The charge flipping method,^47^ as implemented in Superflip,^48^ was used for structure solution, and full matrix least-squares refinement on *F*^2^ was carried out using the programs SHELXTL^49^ and JANA2006.^50^

Under-occupancies were checked at all atomic positions, but only the Mg position shows significant defects, indicating non-stoichiometry in the subcell. All atoms were refined with rather low displacement parameters. The origin of such anomalous thermal behavior remains unknown. Similar anomalous behavior in the Mg_2_PtSi analogue was assigned to the severe absorption problem.^42^ Because of the excellent crystalline quality of the product as evidence in a powder pattern, dynamic effects are probably strong, and they may explain the anomalous thermal behavior as well. If the Mg atoms are refined isotropically, some improvement of the thermal behavior of all heavier atoms in the system results, with some over-occupancy on Mg1 (site 2*b*). When an additional spherical absorption correction is applied to the data, the anomalous thermal behaviors are suppressed, and all the atomic positions are fully occupied.

As for Mg_2_PtSi,^42^ the abnormal thermal behavior can also be corrected by refining the anomalous scattering coefficients *f*′ and *f*″ using the program Jana2006.^50^ Another possible explanation for this unusual thermal behavior can be ascribed to complex twinning in the non-centrosymmetric space group and perhaps the domain corresponding to an orthorhombic distortion to the space group *Cmc*2_1_.^51,52^ However, the refinement of the structure in the orthorhombic setting results in slightly more reasonable displacement parameters but also higher residuals. Atomic positions and labels were standardized using the program STRUCTURE TIDY.^53^ Crystal data, data collection, and structure refinement details are summarized in Table 1, and Table 2 contains the atomic positions and equivalent displacement parameters. Further details of the crystal structure investigations (CIF file) may be obtained from Fachinformationszentrum Karlsruhe, 76344 Eggenstein-Leopoldshafen, Germany (fax: (+49)7247-808-666; e-mail: crysdata@fiz-karlsruhe.de) on quoting the deposition number CSD 1941262.

**Table 1:** Crystal Data and Structure Refinement Details for Mg_7_Pt_4_Ge_4_

empirical formula	Mg_7_Pt_4_Ge_4_
formula weight	1240.89
temperature	293(2) K
wavelength	0.71073 Å
crystal system	hexagonal
space group	*P*6_3_*mc* (186)
unit cell dimensions	*a* = 8.5943(1) Å
	*c* = 8.4496(4) Å
CSD number	1941262
volume	540.5(1) Å^3^, *Z* = 2
density (calculated)	7.625 g/cm^3^
absorption coefficient	62.86 mm^–1^
*F*(000)	1048
theta range for data collection	3.65 to 29.1°
index ranges	–11 ≤ *h* ≤ 10, –11 ≤ *k* ≤ 11, –10 ≤ *l* ≤ 10
reflections collected	4107 [*R*σ = 0.031]
independent reflections	544 [*R*_int_ = 0.058]
observed reflections	505
refinement method	full-matrix least squares on *F*^2^
refinement program	JANA2006
data/restraints/parameters	544/1/36
goodness-of-fit	1.470
flack parameter	–0.01(2)
final *R* indices [*I* > 2sigma(*I*)]	*R*_1_ = 0.0266, w*R*_2_ = 0.0747
*R* indices (all data)	*R*_1_ = 0.0293, w*R*_2_ = 0.0754
extinction coefficient	0.0019(2)
largest diff. peak and hole	1.126 and –1.630 e Å^–3^

**Table 2:** Fractional Atomic Coordinates and Equivalent Isotropic Displacement Parameters (/Å^2^) of Mg_7_Pt_4_Ge_4_

atoms	Wyckoff pos.	*x*	*y*	*z*	*U*_eq_
Mg1	2*b*	1/3	2/3	0.400(2)	0.014(5)
Mg2	6*c*	0.8439(8)	*x̅*	0.784(2)	0.015(3)
Mg3	6*c*	0.2012(8)	2*x*	0.642(2)	0.023(2)
Pt1	6*c*	0.8361(1)	*x̅*	0.459(2)	0.014(1)
Pt2	2*b*	1/3	2/3	0.752(2)	0.013(1)
Ge1	2*a*	0	0	0.000(2)	0.017(2)
Ge2	6*c*	0.5040(3)	*x̅*	0.722(4)	0.015(1)

### Electronic-Structure Calculations

The electronic structures of the title compound Mg_7_Pt_4_Ge_4_ (I), the hypothetical defect-free “Mg_2_PtGe” phase (II), and the binary Mg_3_Pt phase (III) were calculated self-consistently using the tight-binding LMTO (TB-LMTO) method within the atomic sphere approximation (ASA) using the LMTO, version 47, program.^54,55^ Exchange and correlation were treated in a local density approximation (LDA).^56^ Relativistic effects were taken into account using a scalar relativistic approximation.^57^ As the investigated structures were not closely packed, overlapping Wigner–Seitz (WS) atomic empty spheres were added with an automatic procedure to create an adequate potential.^58^ Six, two, and five sets of empty spheres were generated for structure I, II, and III, respectively. The WS radii of all empty spheres ranged from 0.6 to 1.04 Å. The basis set included Mg-3s/3p/, Pt-6s/6p/5d/(4f), and Ge-4s/4p/(3d) and E-1s/(2p) orbitals for the empty spheres (down-folded orbitals in parentheses).^59^ The reciprocal space integrations to determine the self-consistent charge density and densities of states (DOS) were performed by the tetrahedron method^60^ using 222, 222, and 305 *k* points, for I, III, and II, respectively, in the irreducible wedges of the corresponding Brillouin zones for the models. Crystal orbital Hamilton population (COHP)^61^ curves and their integrated values (iCOHP) were used to analyze relative bond strengths via orbital interactions. All empty sphere orbitals were down-folded before running COHP calculations. Since the COHP is an energy partitioning, negative/positive values indicate bonding/antibonding interactions. The Fermi level in all figures is taken as the zero energy level, and the COHP curves are drawn by reversing their values with respect to the energy scale (i.e., −COHP vs E). Hence, the calculated peak values become negative for antibonding and positive for bonding interactions.

### Magnetic Measurements

The evolution of the magnetic susceptibility with the temperature χ(*T*) was measured on a physical properties measurement system (PPMS; Quantum Design, USA). Polycrystalline samples were loaded onto polypropylene capsules, which were mounted on a brass sample holder. The magnetization signals of the title compounds are magnitudes larger than that of the empty sample holder. Therefore, no diamagnetic corrects were applied.

## Results and Discussion

### Synthesis and Crystal Structure

High-temperature reaction of the elements in a 2:1:1 ratio yielded single crystals of Mg_2–*x*_PtGe in a trigonal prismatic shape (Figure S1 in the Supporting Information). The X-ray powder diffraction pattern (Figure S1 in the Supporting Information) of samples with nominal composition “Mg_2_PtGe” showed sharp diffraction peaks and only a very weak background, indicating a high crystalline quality of the reaction product. The main peaks correspond to the expected Mg_2_PtSi-type cell, but some satellite reflections indicate a superstructure. This is confirmed by single crystal diffraction (SCXRD) investigation of a crystal grown with a 50% Mg excess (i.e., with a Mg/Pt/Ge ratio of 3:1:1), for which rather weak superstructure reflections were disregarded at first approximation. A structure solution in the space group *P*6_3_/*mmc* for Mg_2_PtSi (Pearson code *hP*8) reveals significant defects at the Mg site, yielding a composition Mg_1.88(6)_PtGe, i.e., Mg_2–*x*_PtGe with *x* = 0.12 (see the Supporting Information). However, elongated thermal ellipsoids for the Ge atoms indicate the violation of one mirror plane perpendicular to the *c*-axis, and SCXRD analysis of another high-quality single crystal obtained under similar reaction conditions clearly revealed after a careful examination of the reciprocal space reconstruction images a commensurate 2 × 2 × 1 superstructure of the Li_2_CuAs-type subcell. A lower space group symmetry, *P*6_3_*mc*, is confirmed by successful refinement, with the composition Mg_2–*x*_PtGe (*x* = 0.25). A full ordering of the Mg vacancies yields a stoichiometric phase Mg_7_Pt_4_Ge_4_, which corresponds to a new structure type, Pearson code *hP*30, Wyckoff sequence *c*^4^*b*^2^*a*. Noteworthy, the previously reported Si analogue, Mg_2_PtSi, was prepared under a high pressure to avoid Mg evaporation, and its Li_2_CuAs type of structure features rigorously planar (PtSi) honeycomb layers. In contrast, the corresponding (PtGe) layers in Mg_7_Pt_4_Ge_4_ are puckered, as the ordering of the Mg defects is followed by a relaxation of the Mg hexagonal diamond-like framework. The whole process generates three symmetrically unrelated Mg positions with direct Mg–Mg connections from three to five (for a bond length cut-off of 3.5 Å). Similar (MgPt) bimetallic 6^3^ layers occur in the binary Mg_3_Pt, in which the Ge atoms are replaced by Mg, resulting in a  ×  × 1 supercell relative to the Mg_2_PtSi structure.

**Figure 1:**
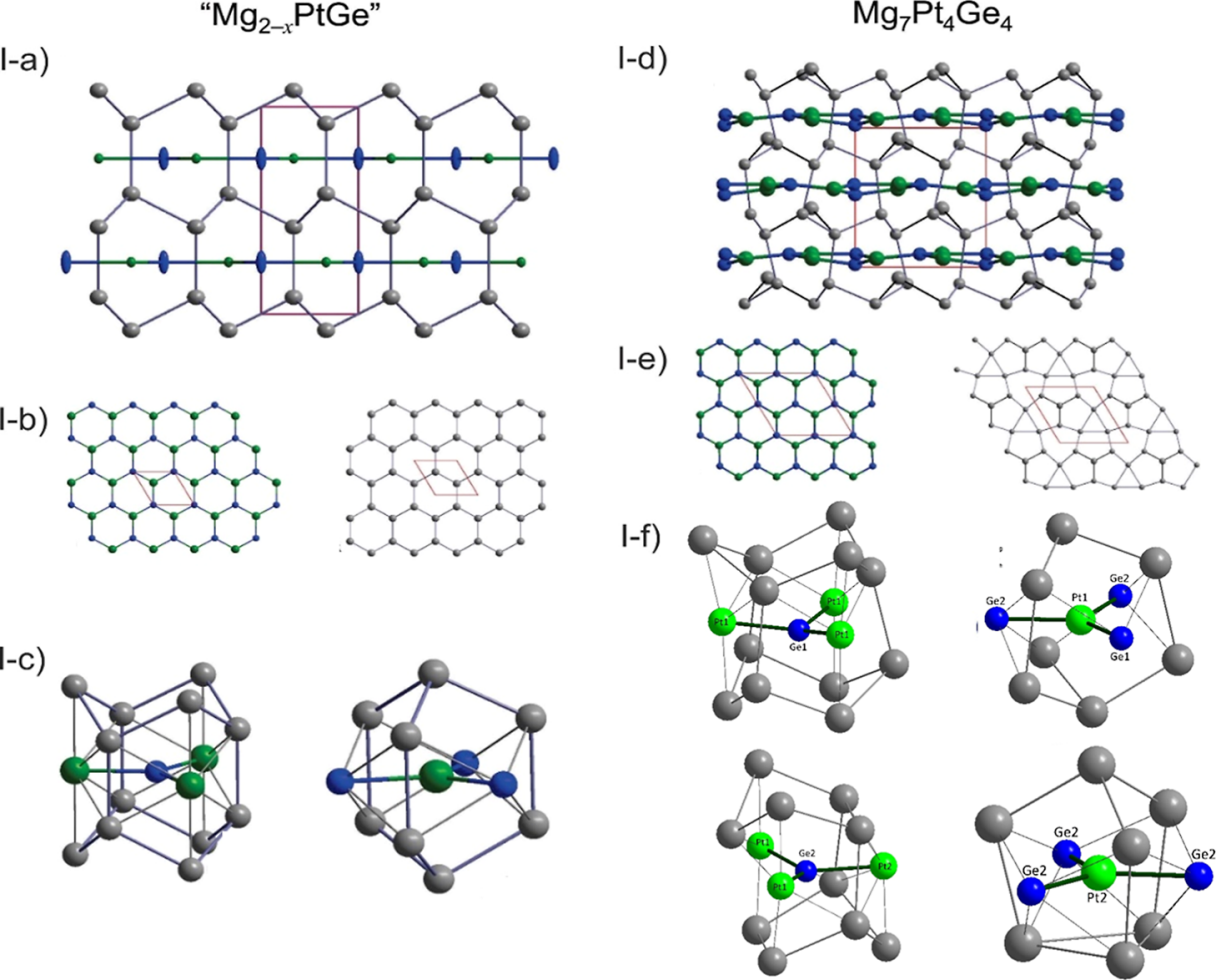
Graphical representations of I(a–c) a Mg_2_PtSi-type subcell and I(d–f) Mg_7_Pt_4_Ge_4_ with the 2 × 2 × 1 supercell. Hexagonal diamond framework of Mg (gray sphere) in (I-a) and its defect derivative in (I-d), apparent when viewed along [010], and the honeycomb layers in the voids consisting of Pt (green spheres) and Ge (blue spheres) planar in (I-b) but puckered in (I-e), view along [001]. Local coordination environments in pristine (I-b) and defective (If) supercells. For more details, see text.

The topology of the defect-free Mg_2_PtSi structure according to the reticular chemistry notation^62^ yields the Mg framework a **dia** net topology, which is penetrated by bi-atomic **hcb** layers of Pt and Si, to form a hetero-dual **lon-d** net (see Figure 1). The Si atoms are located in hexagonal prismatic cavities, and the Pt atoms are located in bi-capped trigonal prismatic cavities of 12 and 8 Mg atoms, respectively. However, in Mg_7_Pt_4_Ge_4_, as 1/8 of the Mg atoms forming the 4*b*-3D framework are missing, the vacancies ordering within the defective Mg framework, alongside non-planar 6^3^ (PtGe) layers, become the distinctive feature. The structural distortion preserves the hexagonal symmetry of the system. However, violations of the mirror perpendicular to the *c*-axis results in a lower space group symmetry. If we consider the Mg covalent radii to be 1.6 Å,^63^ the complex 3D network of Mg in Mg_7_Pt_4_Ge_4_ consists of three connected Mg1,3 and four connected Mg2. Two additional Mg3–Mg3 interactions at longer 3.403 Å yield triangular rings of Mg3, and these result in five Mg–Mg connections for each Mg3 site. The defective tetrahedral Mg framework of Mg_7_Pt_4_Ge_4_ involves two symmetry equivalent corrugated nets at *z* = 1/4 and *z* = 3/4, which are related to each other by a glide mirror plane and interconnected along the *c*-axis. As listed in Table 3, the Mg–Mg shortest distances within the planes are shorter than the sum of covalent radii and increase in the order Mg2–Mg3 < Mg1–Mg2. The interconnection between the layers is realized through slightly longer Mg2–Mg3 (3.094 Å). For comparison, the Mg–Mg distance in binary Mg_2_Ge is 3.19 Å, very close to the sum of covalent radii.^63^

**Table 3:** Selected Interatomic Distances and iCOPH (/eV) Values for Mg_7_Pt_4_Ge_4_

atom pairs		distances (Å)	–iCOHP	atom pairs		distances (Å)	–iCOHP
Mg1/Pt2:		2.75(2)	1.28	Pt1/Ge1:		2.465(2)	2.68
Mg1/Pt1:	3×	2.785(9)	0.96	Pt1/Ge2:	2×	2.487(3)	2.63
Mg1/Ge2:	3×	2.82(2)	0.92	Pt2/Ge2:	3×	2.540(5)	2.44
Mg1/Ge2:	3×	3.75(2)	0.07	Mg3/Pt2:		2.69(1)	1.32
Mg2/Pt1:		2.7444)	1.31	Mg3/Pt1:		2.75(1)	1.35
Mg2/Pt1:	2×	2.809(9)	0.99	Mg3/Ge2:	2×	2.928(8)	0.78
Mg2/Ge2:	2×	2.862(8)	0.86	Mg3/Pt1:	2×	3.159(9)	0.55
Mg2/Pt2:		2.89(2)	0.88	Mg3/Ge1:		3.22(1)	0.39
Mg2/Ge1:		2.96(4)	0.71	Mg3/Ge2:	2×	3.292(9)	0.37
Mg2/Ge1:		3.34(4)	0.28	Mg1/Mg2:	3×	3.06(1)	0.54
Mg2/Ge2:	2×	3.93(2)	0.04	Mg2/Mg3:	2×	2.98(1)	0.59
Mg3/Mg3:	2×	3.40(1)	0.29	Mg2/Mg3:		3.09(2)	0.28

In the Mg_2_PtSi-type subcell of Mg_2–*x*_PtGe, the tetrahedral Mg framework also involves two puckered honeycomb nets, but ecliptically stacked at *z* = 0 and *z* = 1/2 connected along the *c*-axis. The Mg–Mg interatomic distances are 2.85 Å for in-layer and 2.90 Å between layers, much shorter than those in the defective Mg_7_Pt_4_Ge_4_ as expected. The Pt–Ge distances (2.51 Å) in the planar 6^3^ layers are close to the sum of covalent radii (1.37 + 1.22 Å), albeit shorter, and corresponding to the average distance observed in Mg_7_Pt_4_Ge_4_ (2.46 to 2.54 Å). Similar Pt–Ge distances are found in Ca_10_Pt_7_Ge_3_ (from 2.46 to 2.58 Å) and are consistent with strong bonds.^35^

The vacancy ordering in Mg_7_Pt_4_Ge_4_ has a direct impact on the local coordination geometries of the different atomic sites, as depicted in Figure 1. The Pt and Ge atoms occupy two crystallographically independent positions, and they differ primarily by their local coordination. The local coordination geometries of Ge1 and Ge2 by Mg atoms both correspond to defective shapes of the hexagonal prismatic cavities originally found in the pristine Mg_2_PtSi structure for the Si sites. Relative to that defect-free phase, the coordination spheres in Mg_7_Pt_4_Ge_4_ are missing either three Mg atoms from the same hexagonal ring for Ge1 sites (2*a*) or two Mg atoms from two distinct opposite rings for Ge2 sites (6*c*). For the Pt atoms which are originally in bi-capped trigonal prismatic cavities in the pristine structure, the Pt1 (site 6*c*) are missing one basal Mg atoms, while Pt2 sites (2*b*) are missing one capping Mg atom, so that the threefold axis is locally preserved for the latter only. In comparison, the Mg–Pt distances are close to those in REMgPt (2.732(6) to 2.739 Å)^63^ and significantly longer than those in Ca_2_MgPt_2_ (2.649(1) Å).^38^

**Figure 2:**
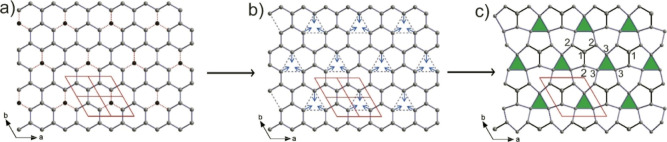
Atomistic model of the Mg vacancy ordering and framework rearrangement from the Mg_2_PtSi-type subcell to the 2 × 2 × 1 superstructure of Mg_7_Pt_4_Ge_4_. The black spheres in (a) represent the positions of the vacancies. The arrows in (b) depict the distortion, and in (c) the Mg(3) position forming triangular rings is highlighted in green.

As shown in Figure 2, the complex Mg framework in Mg_7_Pt_4_Ge_4_ can be easily derived from the diamond-like Mg framework of the Mg_2_PtSi parent structure.^64^ In the latter, the nonplanar 6^3^ Mg layers of condensed hexahedral rings in the *ab* plane with a chair conformation are formed by interconnecting parallel rows of Mg zigzag chains running in the *a*-direction. Since 1/8 of the Mg positions are vacant in Mg_7_Pt_4_Ge_4_, an ordering of the vacancies proceeds by the removal of every 4th Mg atoms in every second row. In Figure 2a, the positions of these Mg vacancies in the pristine Mg_2_PtSi are indicated by black spheres. After their removal, the three Mg atoms in the vicinity of the vacancies are shifted toward their center to form the triangular rings observed in the structure of Mg_7_Pt_4_Ge_4_, as illustrated in Figure 2b. These displacements trigger a distortion of the remaining hexagonal rings, and the overall process preserves the 6_3_-screw axis. A similar distortion of the hexagonal rings within the Au tetrahedral framework is observed in the series Ae_*m*_[E_3_]_*n*_Au_2(*m*+*n*)_ due to vacancies in the layers.^18^ For Mg_7_Pt_4_Ge_4_, the resulting puckered Mg layers are formed by a complex network of three-, five-, and six-membered rings, which are condensed by sharing edges (Figure 2c). These complex (3.5.6.5)_3_(5^2^.6)_3_5^3^ nets are subsequently interconnected along the *c*-axis to form a 3D framework of Mg atoms, with voids occupied by Pt and Ge atoms forming a 6^3^ puckered honeycomb layer with Pt connected to three Ge atoms and vice versa. Similar defective, but not relaxed, beehive-like sheets made from alternating Zn and As atoms with vacancies are described in the [Zn_2_As_3_]^5–^ sub-structure of the Zintl phase Eu_11_Zn_4_Sn_2_As_12_.^65^

**Figure 3:**
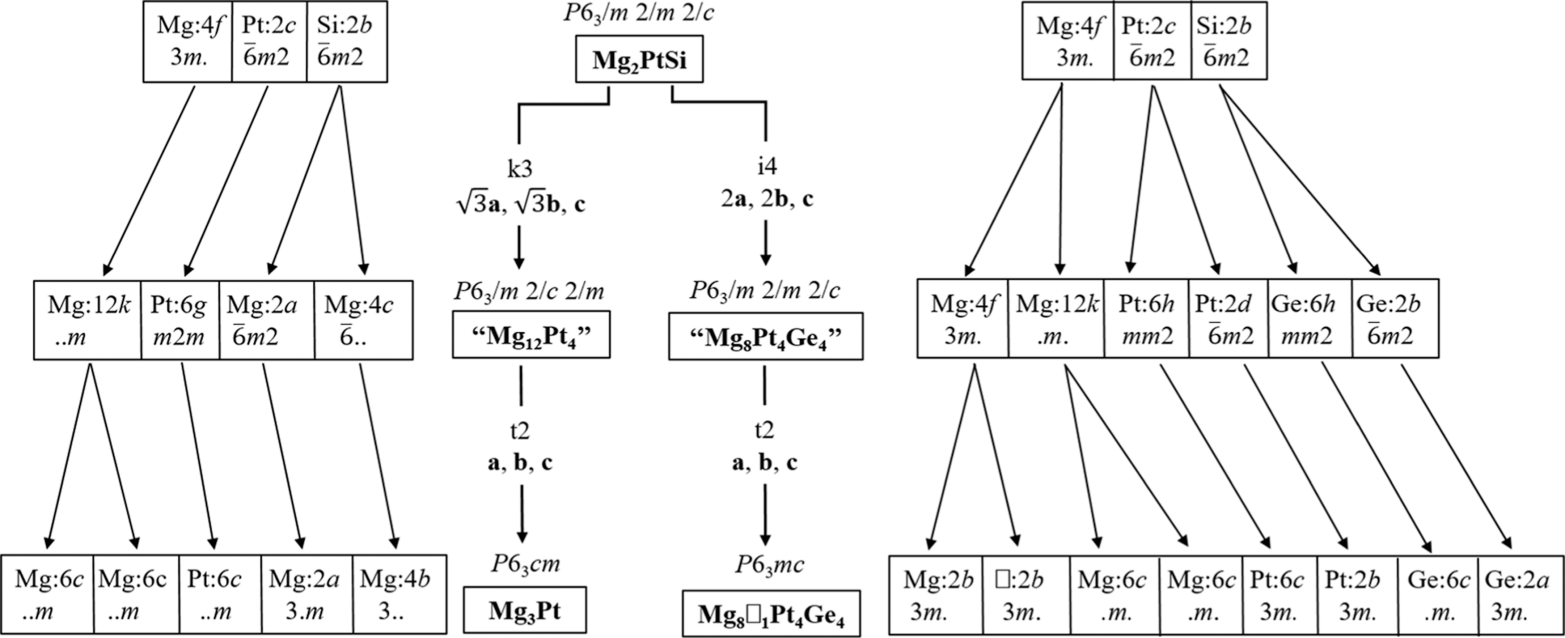
Group–subgroup trees of the transformations from the aristotype Mg_2_PtSi (space group *P*6_3_/*mmc*) to the daughter structures Mg_3_Pt (*P*6_3_*cm*) and Mg_7_Pt_4_Ge_4_ (*P*6_3_*mc*).

Mg_2_PtSi (Li_2_CuAs-type) is the aristotype structure of the binary Mg_3_Pt (Cu_3_P type). The latter can be derived from the former by the replacement of one Si atom in the 6^3^ layers by a Mg atom. This is followed by a distortion of the (4*b*-3D) diamond-like framework of Mg atoms likely due to mutually exclusive interactions between Mg–Pt (within the 6^3^ layers) and Mg–Mg (between 6^3^ layers and diamond framework), leading to some kind of chemical frustration. Group–subgroup trees between the aristotype Mg_2_PtSi structure and the two daughter structures of Mg_3_Pt and Mg_7_Pt_4_Ge_4_ can be constructed as shown in Figure 3. First, the Mg_2_PtSi structure is subjected to an isomorphic transformation of index 4 (i4) in the supercell (2**a**, 2**b**, **c**) to yield the hypothetical defect-free phase “Mg_8_Pt_4_Ge_4_” in the same space group *P*6_3_/*mmc*. Then, a *translationengleiche* transformation of index 2 (t2) generates the subgroup (*P*6_3_*mc*) by removing the *m* mirror perpendicular to the *c*-axis. This allows the splitting of one Mg occupied 4*f* site into two 2*b* sites, in which one is occupied by Mg and the other is vacant. Alternatively, starting from the aristotype Mg_2_PtSi structure, a *klassengleiche* transformation of index 3 (k3) in the supercell (**a**, **b**, **c**) generates the hypothetical “Mg_12_Pt_4_” (*P*6_3_/*mcm*), in which the splitting of one Si (2*b*) positions generates two sites (2*a* and 4*c*), all occupied by Mg atoms. A subsequent *translationengleiche* transformation of index 2 (t2) along with a symmetry reduction to *P*6_3_*mc* is due to the distortion of the 4b-3D diamond-like Mg lattice destroying the mirror *m* perpendicular to the 6_3_-screw axis, resulting in the splitting of one Mg (12*k*) position into two Mg (6*c*) positions.

The Li_2_CuAs-type structure of the aristotype Mg_2_PtSi structure represents the hexagonal alternative to the cubic (inverse) Heusler-type structure.^66,67^ The low-temperature modification of the polymorphic phase Na_2_MgPb is of hexagonal Li_2_CuAs type. At high temperatures, it transforms into a cubic inverse Heusler type (Li_2_AgSb type).^66^ However, factors that determine the structural selection between cubic Heusler and the hexagonal rival, the Li_2_CuAs type, remain poorly understood. As a general trend, compounds of heavier congener prefer the cubic Heusler. Interestingly, an unprecedented tetragonal superstructure of the inverse Heusler has also been reported recently in Mn_2–*x*_PtSn.^11^ The Li_2_CuAs type also belongs to a larger family of hexagonal structures (including the prominent AlB_2_ type), which are defined by an interpenetration of a four-bonded three-dimensional (4*b*-3D) diamond-like framework and 2D graphite-type (6^3^) planar layers of condensed hexagons. Some orthorhombic derivatives are known, like the YPd_2_Si-type structure (space group *Pnma*), in which the noble metal Pd forms the 4*b*-3D tetrahedral framework, while Y and Si atoms are located in the 6^3^ planar layers.^68^ In the series with the general formula Ae_*m*_[E_3_]_*n*_Au_2(*m*+*n*)_ (Ae = alkaline earth and E = triel or tetrel), Au atoms form the 4*b*-3D net and triangular E_3_ units encapsulated within the distorted hexagonal prismatic cavities of the Au-framework structures.^3,18^ This structure series may be viewed as further defect variants of the structure family with a interlocked tetrahedral framework and honeycomb layers, where the defects are located rather within the 6^3^ layers and with different types of vacancy ordering endowing high versatility to the system.

The flexibility in the composition of the cubic (inverse) Heusler systems and related structures is ubiquitous. These systems often attain a stable valence balanced composition by accommodating large defect concentrations, opening up multiple dimensions for the discovery of multicomponent defective structures based on intrinsic and extrinsic defects which compensate for the nominally non-18-electron count of the structure.^5^ However, for the hexagonal rival, Li_2_CuAs type, non-stoichiometry is rather seldom.^66,67^ Therefore, the defect formation in the 18-valence electron system Mg_2_PtSi is considered merely the result of Mg evaporation at high temperatures, but this does not explain the vacancy ordering in defective Mg_2–*x*_PtGe. It is therefore of interest to identify other possible factors behind the large defect formation in the title compound, and in this respect, the electronic band structure may provide some valuable clues.^70^

### Electronic Structure and Bonding Analysis

**Figure 4:**
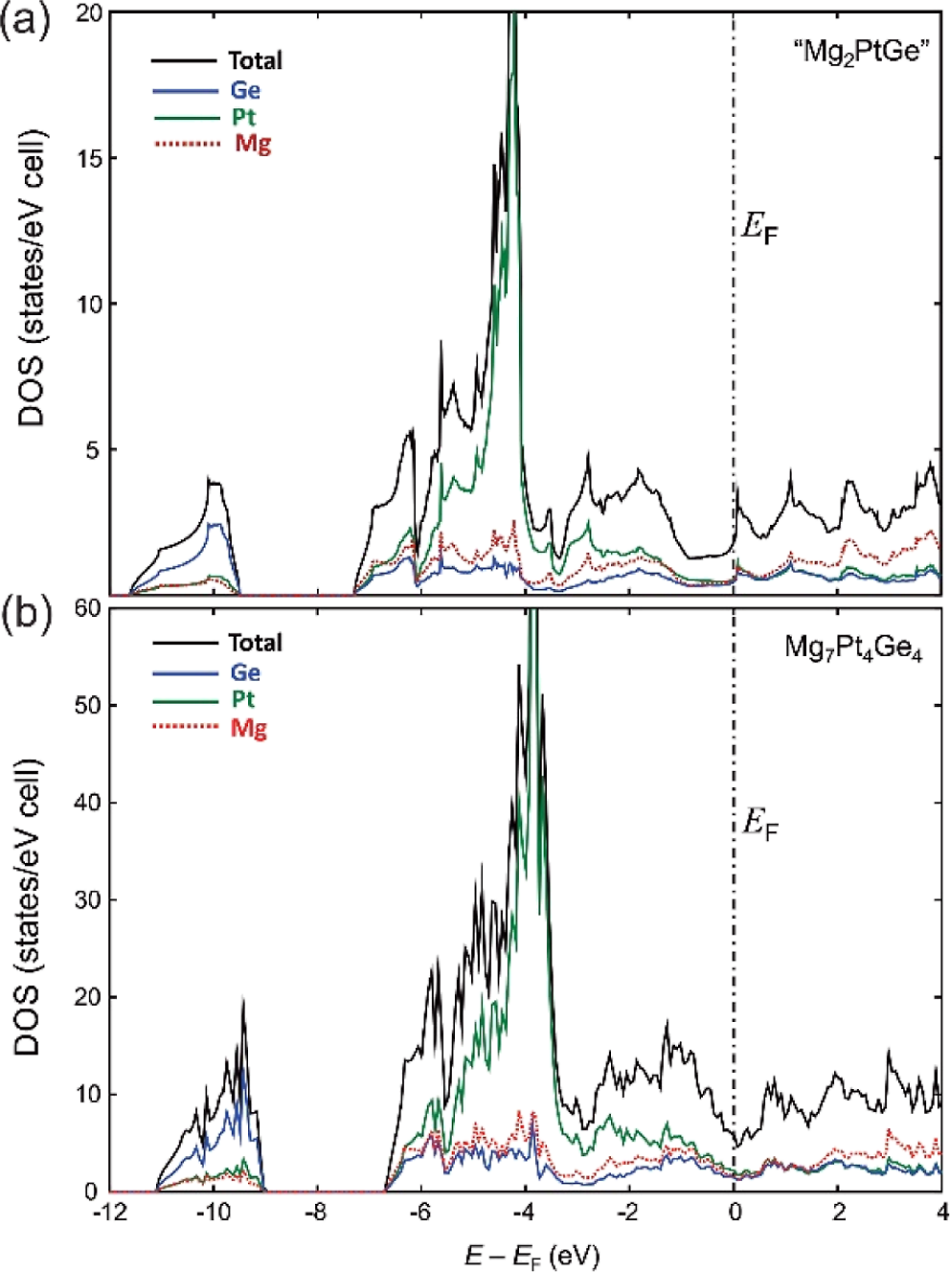
DOS curves and the individual atomic contributions obtained from LMTO calculations on (a) hypothetical vacancy-free “Mg_2_PtGe” and (b) observed Mg_7_Pt_4_Ge_4_ phase in the 2 × 2 × 1 supercell.

The closely related crystal structures of hypothetical defect-free “Mg_2_PtGe” and experimentally obtained Mg_7_Pt_4_Ge_4_ translate into similar DOS curves obtained from LMTO calculations (Figure 4). The narrow region in the DOS curves at the bottom of the energy scale (around −10 eV) is mainly contributed by the Ge-4s orbital, suggesting negligible sp hybridization of the Ge atoms in the systems. Toward higher energies, this region is followed by a rather broad one formed by s and p states from the active metal Mg and the late main group element Ge in combination with Pt 5d states. Similar to Au,^18^ the valence states of Pt are strongly subject to relativistic effects.^26^ As a consequence, a strong hybridization of these states is observed (see also Figures S6 and S7 in the Supporting Information). Above the Fermi level, the contribution of the more electropositive Mg to the DOS becomes dominant, confirming a charge transfer from Mg to the Pt/Ge sub-structure, rendering it anionic. However, even the contribution of Mg to states below the Fermi level is significant, implying a substantial participation of the Mg atoms in the covalent bonding of the systems, showing significant electron back-donation from the anionic substructure due to the relatively strongly polarizing nature of Mg cations. The occurrence of a deep pseudo-gap near the corresponding Fermi levels (*E*_F_) reveals for both hypothetical “Mg_2_PtGe” and Mg_7_Pt_4_Ge_4_ points to deviations from the free-electron like behavior. In comparison, the calculated band structure of Mg_3_Pt shows enhanced free-electron-like characteristics with a total absence of a pseudo-gap near *E*_F_. Instead, the DOS curve (see Figure S8 in the Supporting Information) is more consistent with an opened valence band system, suggesting that the compound is electron deficient. For “Mg_2_PtGe”, the pseudo-gap in the DOS curves is located below the Fermi level (Figure 4a). Such a feature is usually associated with an instability in the electronic structure. In contrast, in Mg_7_Pt_4_Ge_4_, it falls into the pseudo-gap (Figure 4b), which is frequently associated with stability. Interestingly, showing such a feature satisfies the 18-electron valence rule.^68^ Here, the 18-electron rule gets violated, yet the feature of moving the Fermi level to the pseudo-gap is followed. In that sense, the defect formation seems to be justified by the electronic band structure, yet it is realized at an unexpected valence electron count. A COHP analysis allows deeper insights into the structure directing factors and, in particular, the origin of the Mg vacancy formation. The overall −COHP curves of the defect-free “Mg_2_PtGe” reveal substantial occupation of states with the antibonding character starting from −1 eV, confirming an excess of valence electrons in this phase. This is rectified in Mg_7_Pt_4_Ge_4_, where the Fermi level ideally marks the separation between bonding and antibonding states. Thus, bonding is optimized upon Mg vacancy formation and the reduction of the valence electron count.

For the imaginary “Mg_2_PtGe” with its 18-valence electron (ve) per formula unit (fu), a similar optimization occurs below *E*_F_ at −1 eV. According to the iDOS, the ve count at −1 eV is roughly 17.3 ve/fu, very close to 17.5 ve/fu of the defective Mg_1.75_PtGe (or 70 ve/fu for Mg_7_Pt_4_Ge_4_). The rigid band approximation can therefore predict the Mg vacancy formation, as a mean to deplete the antibonding states. Interestingly, the formation of the Mg defect is also observed in the Si analogue prepared at a normal pressure, but not described. To obtain stoichiometric Mg_2_PtSi, high-pressure high-temperature synthesis is used, arguably to avoid Mg volatility.^42^ Our findings here suggest that the Mg deficiency is rather driven by the system’s desire to optimize its chemical bonding.

In contrast to the ternary systems, the COHP curves for Mg_3_Pt feature plenty of bonding states around the Fermi level (see Figure S8 in the Supporting Information), confirming that the phase is electron deficient and is able to accommodate more electrons, which could be the reason for its “hydrogen pump effect”. In fact, all the bonding states should be filled around 1.8 eV above *E*_F_. According to iDOS, the electron count at 1.8 eV is 108 ve per cell (*Z* = 6) as expected from the 18 ve rule.

**Figure 5:**
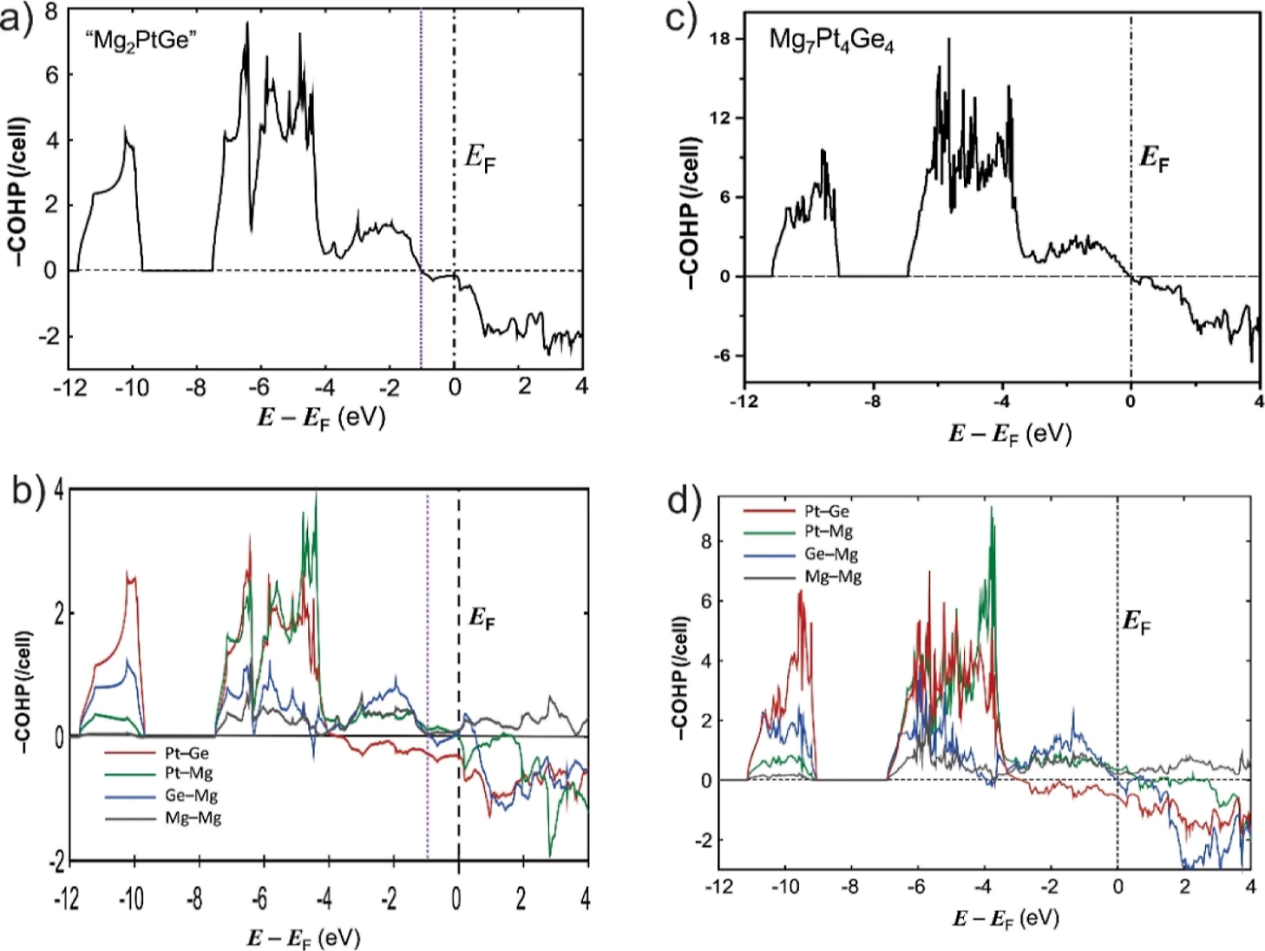
Cumulated COHP curves for all interactions (cut-off 3.5 Å) (a,c) and for selected interactions (b,d) in the hypothetical vacancy-free “Mg_2_PtGe” phase (a,b), and the experimental Mg_7_Pt_4_Ge_4_ in the 2 × 2 × 1 supercell (c,d).

Looking at individual interactions, the cumulative COHP curves of all Pt–Ge contacts in both “Mg_2_PtGe” and Mg_7_Pt_4_Ge_4_ exhibit significant filling of antibonding states starting deep below *E*_F_ of around −4 eV and expanding up to above the Fermi level (Figure 5). Those antibonding interactions arise from Pt–Ge interactions and add for “Mg_2_PtGe” to the electronic destabilization just below the Fermi level. The corresponding states are emptied in Mg-deficient Mg_7_Pt_4_Ge_4_, for which the Mg–Pt and Mg–Ge interactions remain strongly bonded up to the Fermi level. Recently, we could identify similar features in the COHP curves of La_7_Co_2_Ge_4_^16^ with the valence band maximum consisting of bonding states from “cation–anion” contacts (La–Co and La–Ge) overlapping and antibonding states from interactions within the anionic network (Co–Ge). This was associated with an electron back-donation from the “anionic” to the “cationic” component through multicenter interactions.^13−16^ Back-donation from the anionic, i.e., Pt–Ge, substructure to Mg is a mechanism to relieve the anti-bonding contribution from Ge–Pt interactions around the Fermi level.

These observations agree well with the −iCOHP values (Table 3). Indeed, in the subcell of defect-free “Mg_2_PtGe”, the Pt–Ge bonds within the anionic layers have the largest −iCOHP values (2.36 eV/bond) in the structure, much larger than that of the next contact Mg–Pt (0.93 eV in average). Unexpectedly, the Mg–Ge bonds (0.76 eV/bond) have strength comparable to Mg–Mg contacts (0.72 eV/bond), suggesting that Mg–Ge bonds may be predominantly ionic. However, the most frequently occurring Mg–Pt contacts in the structure have the largest contribution to the total iCOHP (31%), albeit roughly comparable with that of the Pt–Ge contacts (29%). The overall Mg–Ge contact contribution is the lowest (19%) but close to that of Mg–Mg contacts (21%). Since Mg is the most electropositive element in the structure, the contribution of Mg–Mg contacts to the total iCOHP is expected to be the lowest. Hence, the high contribution of the Mg–Mg contacts in the covalent bonding of “Mg_2_PtGe” is very unusual and may represent a fingerprint of valence electron excess, retained in the “cationic” sub-structure. For comparison, in the previously described Ca_2_MgPt_2_, the Ca–Pt bonds (0.75 eV/bond) are less covalent than Mg–Pt bonds (1.54 eV/bond) and Ca–Ca interactions contribute to less than 5% of the total iCOHP. In Ca_2_Pt_2_Ge, Pt–Ge contacts within the anionic sub-structure contribute to 47% of the total iCOHP and Pt–Pt 17%, while Ca–Pt 21%, Ca–Ge 14%, and Ca–Ca less than 1%.^39^

As the Mg vacancies are formed in the Mg_7_Pt_4_Ge_4_ structure, similar trends in the relative bond strength are retained, but with the strongest Pt–Ge contacts (2.63 eV/bond in average) now having an overall contribution of 31% to the total Hamilton population, while the Mg–Pt contacts (1.13 eV/bond on average) sharply increase to 51%. Meanwhile, the overall contribution of the Mg–Ge contacts decreases to 11%, still roughly close to that of Mg–Mg contacts, which is the lowest at 8%. It appears that relative to the defect-free phase “Mg_2_PtGe”, the covalent character of Pt–Ge and Pt–Mg contacts increases upon defect formation, while the Mg–Mg and Mg–Ge contacts become significantly less covalent. Thus, in Mg_7_Pt_4_Ge_4_, the covalent bonding system consists mainly of multicenter Mg–Pt bonds followed by stronger two-center Pt–Ge bonds, while Mg–Mg and Mg–Ge interactions are mainly ionic.

At first glance, Mg_2_PtSi (18 ve/fu) nicely fits the Zintl–Klemm concept, as would “Mg_2_PtGe” according to 2Mg^2+^, 1T^–^ (T = Si, Ge; 3-bonded), 1 Pt^3–^ (pseudo Tl atom). However, the charge assignment is purely formal and significant covalent bonding character is expected between the cationic Mg and the anionic (PtSi) partial structures. Similar trigonal planar coordinations of Pt and Si/Ge are found in the anionic sub-structure of Ca_10_Pt_7_Tt_3_ (Tt = Si, Ge),^42,43^ for which the Zintl–Klemm concept was successfully applied to describe the chemical bonding by assuming the pseudo-main group behavior of negatively polarized Pt atoms. In Ca_10_Pt_7_Si_3_, Si and Pt atoms are sp^2^ hybridized, leading to Pt–Ge σ bonds involving Pt 6s and 6p, while the Pt 5d orbitals are nonbonding.^43^ Despite similar local coordination geometry, the bonding picture in hypothetical “Mg_2_PtGe” and defective Mg_2–*x*_PtGe seems to be radically different and cannot be rationalized by the Zintl–Klemm concept. Rather, the chemical bonding of the imaginary defect-free “Mg_2_PtGe” shows the same complexity found in the cubic binary Mg_2_Tt (Tt = Si, Ge, Sn)^69^ family, whose anti-fluorite structure is rather close to the cubic Heusler structure. In Mg_2–*x*_PtGe, the valence electrons are almost equally distributed between Mg–Pt and Pt–Ge bonds. The Pt 5d orbital contribution to the system of covalent bonding is rather significant, which is different to the situation in Ca_10_Pt_7_Ge_3_.^37,38^ It is therefore clear that multicenter bonds involving mainly Mg–Pt contacts are a peculiar trait of the covalent bonding picture in the title compound. This is probably due to the more polarizing Mg cation as compared to a larger Ca cation. For Ca compounds like Ca_5_Ge_3_, Ca-d orbitals are also involved in the covalent bonding system but as an electron acceptor and without affecting the valence electron count (vec).^13^ Other bonding scenarios have been described for Ca_5_MgAgGe_5_^71^ and Ca_4_Ag_2+*x*_Ge_4–*x*_ (*x* = 1/2),^72^ where the Ag/Ge mixing at one Ge position is also in disagreement with the Zintl–Klemm concept due to a conflict with empirically established “structure-directing rules”.

To characterize further the Pt d orbital participation in the covalent bonding of the title compound, the fat band analysis of the band dispersion is used. In this approach, the widths of the bands show the contribution of selected atomic orbitals. Many steep bands crossing the Fermi level show significant contribution from Pt d orbitals (see Figure S7 in the Supporting Information). This seems to confirm that the violation of the 18–*n* valence electron counting rule by the title compound is due to the combined effects of the strong polarizing power of the Mg cation and strong relativistic effects in Pt, which results in the expansion of its d orbitals, leading to an enhanced covalent character of the Pt–Mg interactions.

Interestingly, a strong Pt d orbital contribution to the bonding in the equiatomic phase MgPtSi (TiNiSi type) was discussed in relation to its superconductivity.^73^ For that reason, we also investigated the magnetic properties of Mg_7_Pt_4_Ge_4_.

### Magnetic Properties

The temperature dependence of the magnetic susceptibility [χ(*T*) data] of Mg_7_Pt_4_Ge_4_ measured in a field of 1 kOe is shown in the Supporting Information (Figure S9). In the temperature range of 50–300 K, Mg_7_Pt_4_Ge_4_ displays extremely weak paramagnetic behavior with a nearly temperature-independent susceptibility of χ = 3.8(4) × 10^–5^ emu mol^–1^. This Pauli regime is consistent with deep pseudo-gap in the calculated electronic structure, predicting the compound to be a poor metallic conductor. No transition to a superconducting state was observed down to 1.9 K.

## Conclusions

The crystal structure of the new ternary phase Mg_7_Pt_4_Ge_4_ has been refined from single crystal X-ray diffraction data. Its structure is the first known 2 × 2 × 1 superstructure of the Li_2_CuAs-type structure featuring an ordering of Mg vacancies. A comparison of the computed electronic band structures of a hypothetical defect-free “Mg_2_PtGe” in the Li_2_CuAs type and the defective Mg_7_Pt_4_Ge_4_ reveals that reducing the Mg content is a means for optimizing chemical bonding in the system, by adjusting the overall electron count and avoiding destabilizing, anti-bonding Mg–Ge interactions. The Mg contribution to the system’s bonding is significant and is translated in significant valence electron back-donation from the (Pt, Ge) anionic network. Quantitatively, the covalent bonding system of Mg_7_Pt_4_Ge_4_ consists mainly of multiatom Mg–Pt–Ge interactions followed by strong Pt–Ge interactions, while Mg–Ge and Mg–Mg interactions are predominantly ionic. The unexpectedly high concentration of Mg vacancies and the resulting violation of the 18 valence electron rule result from the combination of the high polarizing power of Mg cations and strong relativistic effect in Pt, with the expansion of the Pt d orbitals, which renders the Pt d-electrons more polarizable.

## Supporting Information Available

Powder diffraction data and structural details of the models used for theoretical calculations (PDF)
