# Supplementary material for: Optimization of Chemical Bonding through Defect Formation and Ordering—The Case of Mg7Pt4Ge4
Source: Inorg Chem. 2023 May 19;62(22):8519–29. doi: 10.1021/acs.inorgchem.2c04312 (PMC10245375; doi:10.1021/acs.inorgchem.2c04312)
Supplement: Supplementary file 1 — ic2c04312_si_001.pdf [file ic2c04312_si_001.pdf]

Supporting Information

Optimization of Chemical Bonding Through Defect Formation and Ordering – the Case of $Mg_7Pt_4Ge_4$

Siméon Ponou,[a,b] Sven Lidin,[b] and Anja-Verena Mudring[a,c]*

[a]) Department of Materials and Environmental Chemistry, Stockholm University, Svante Arrhenius väg 16C, 114 18 Stockholm, Sweden. E-mail: anja-verena.mudring@mmk.su.se
[b]) Centre for Analysis and Synthesis, Department of Chemistry, Lund University, Naturvetarvägen 14, Box 124, SE-22100 Lund, Sweden.
[c]) intelligent Advanced Materials Group, Department of Biological and Chemical Engineering and iNANO, Aarhus University, Åbogade 40, 8200 Aarhus N, Denmark. E-mail: anja-verena.mudring@bce.au.dk

Table of contents

S1. Synthesis and Characterisation

Figure S1. Optical microscope images of a representative $Mg_7Pt_4Ge_4$ sample showing the typical trigonal prismatic single crystal shape.

Figure S2. Experimental and simulated (hexagonal $P6_3mc$) powder pattern for $Mg_7Pt_4Ge_4$.

S2. Structural Details

Table S1. Crystal data and structure refinement details for $Mg_{2-x}PtGe$ (x = 0.12) in the basic Li_2CuAs type unit cell (adopted by Mg_2PtSi), and binary Mg_3Pt.

Table S2. Fractional atomic coordinates and equivalent isotropic displacement parameters (Å^2) for $Mg_{2-x}PtGe$ (x = 0.12).

Table S3. Fractional atomic coordinates and equivalent isotropic displacement parameters (Å^2) for Mg_3Pt.

Figure S3. Atomistic modelling of Mg defect formation and ordering in the superstructure $Mg_7Pt_4Ge_4$ ($P6_3mc$) starting from the simpler Mg_2PtGe ($P6_3/mmc$).

Figure S4. Comparative view of the distorted Mg honeycomb layers in the binary Mg_3Pt (left) and the defective $Mg_7Pt_4Ge_4$ (right) structures.

Figure S5. Similarities and differences between binary Mg_3Pt space group (top) and its ternary derivative $Mg_7Pt_4Ge_4$ (bottom).

S3. Electronic-Structure Calculations.

Figure S6. Projected DOS for Pt atoms, showing significant hybridization of Pt s-d-p atomic orbitals.

Table S4. Selected interatomic distances and ICOPH values for hypothetical defect-free "Mg_2PtGe".

Figure S7. Fat band representation of the Pt 5d contribution to the band structure of hypothetical "Mg_2PtGe" ($P6_3/mmc$).

Figure S8. COHP plot of selected interactions in Mg_3Pt. All bonding levels will be occupied roughly at 2 eV above E_F, which according to IDOS corresponds to (109 ve/cell) 18,1666 ve/f.u, significantly different from Mg_2PtSi and $Mg_7Pt_4Ge_4$.

S4. Magnetic Susceptibility

Figure S9. Magnetisation as a function of temperature for $Mg_7Pt_4Ge_4$ (polycrystalline) on cooling, measured under dc field of 1T.

S1. Synthesis and Characterisation

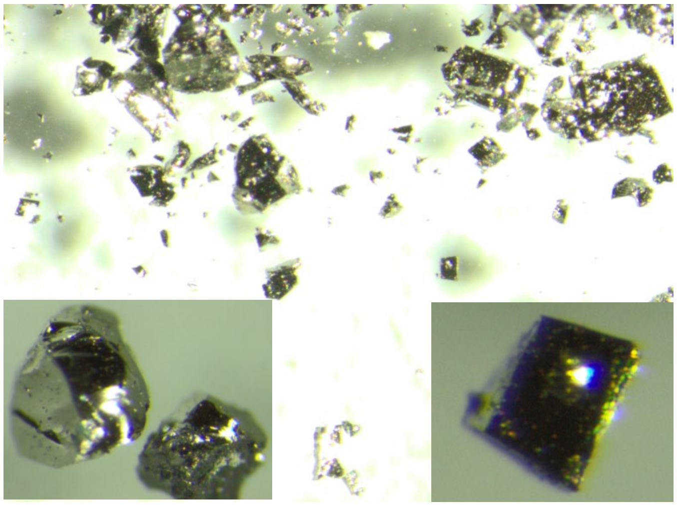

Figure S1. Optical microscope images of a representative $Mg_7Pt_4Ge_4$ sample showing the typical trigonal prismatic single crystal shape.

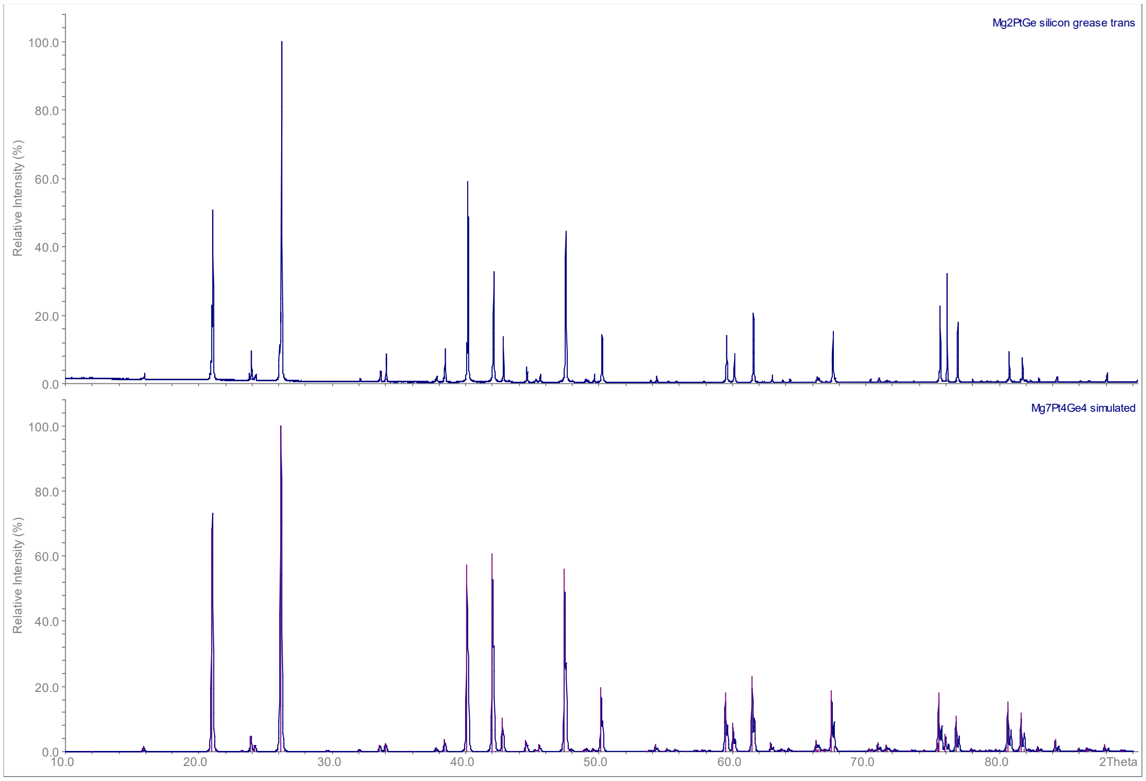

Figure S2. Experimental and simulated (hexagonal $P6_3mc$) powder pattern for $Mg_7Pt_4Ge_4$.

S2. Structural details

Table S1. Crystal data and structure refinement details for $Mg_{2-x}PtGe$ (x = 0.12) in the basic Li_2CuAs type unit cell (adopted by Mg_2PtSi), and binary Mg_3Pt.

Empirical formula	$Mg_{1.88}PtGe$	Mg_3Pt
Formula weight	313.5	268
Temperature	293(2) K	293(2) K
Wavelength	0.71073 Å	0.71073 Å
Crystal system	hexagonal	hexagonal
Space group	$P6_3/mmc$ (194)	$P6_3cm$ (185)
Unit cell dimensions	a = 4.341(3) Å<br>c = 8.509(6) Å	7.9349(7)<br>8.3231(5)
Volume	138.89(16) Å^3, Z = 2	453.84(6), Z = 6
Density (calculated)	7.4955 g/cm^3	5.8834
Absorption coefficient	61.19 mm^{-1}	46.662
F(000)	264	684
Theta range for data collection	4.79 to 28.83°	2.96 to 33.54
Index ranges	$-4 \leq h \leq 5$, $-5 \leq k \leq 4$, $-11 \leq l \leq 10$	$-11 \leq h,k \leq 12$; $-12 \leq l \leq 12$
Reflections collected	706 [R_σ = 0.0089]	6843 [R_σ = 0.0358]
Independent reflections	92 [R_{int} = 0.1116]	618 [R_{int} = 0.0622]
Observed reflections	91	537
Refinement method	Full-matrix least-squares on F^2	
Refinement program	JANA2006	JANA2006
Data / restraints / parameters	92 / 1 / 10	618 / 0 / 27
Goodness-of-fit	2.41	0.97
Flack parameter	n/a	0.55(2)
Final R indices [I>2sigma(I)]	R_1 = 0.0335, wR_2 = 0.0573	R_1 = 0.0226, wR_2 = 0.0484
R indices (all data)	R_1 = 0.0345, wR_2 = 0.0574	R_1 = 0.0262, wR_2 = 0.0514
Extinction coefficient	22(2)	840(30)
Largest diff. peak and hole	2.37 and –2.40 e.Å^{-3}	2.11 and –1.71 e.Å^{-3}

Table S2. Fractional atomic coordinates and equivalent isotropic displacement parameters (Å^2) for $Mg_{2-x}PtGe$ (x = 0.12).

**Atom**	***Wyck.***	**S.O.F.**	***x***	***y***	***z***	**U [/Å^2]**
Pt1	2*c*	1	1/3	2/3	1/4	0.0360(4)
Ge1	2*b*	1	0	0	1/4	0.0743(13)
Mg1	4*f*	0.92(6)	2/3	1/3	0.07985	0.088(4)

Table S3. Fractional atomic coordinates and equivalent isotropic displacement parameters (Å^2) for Mg_3Pt.

Atom	***Wyck.***	***Site***	***x/a***	***y/b***	***z/c***	**U** [/Å^2]
Pt1	6*c*	..*m*	0.67160	0	0.33230	0.0056(1)
Mg1	6*c*	..*m*	0.28491	0	0.50496	0.011(1)
Mg2	6*c*	..*m*	0.62576	0	0.66104	0.014(1)
Mg3	4*b*	3..	2/3	1/3	0.37286	0.013(1)
Mg4	2*a*	3.*m*	0.00000	0	0.26965	0.010(1)

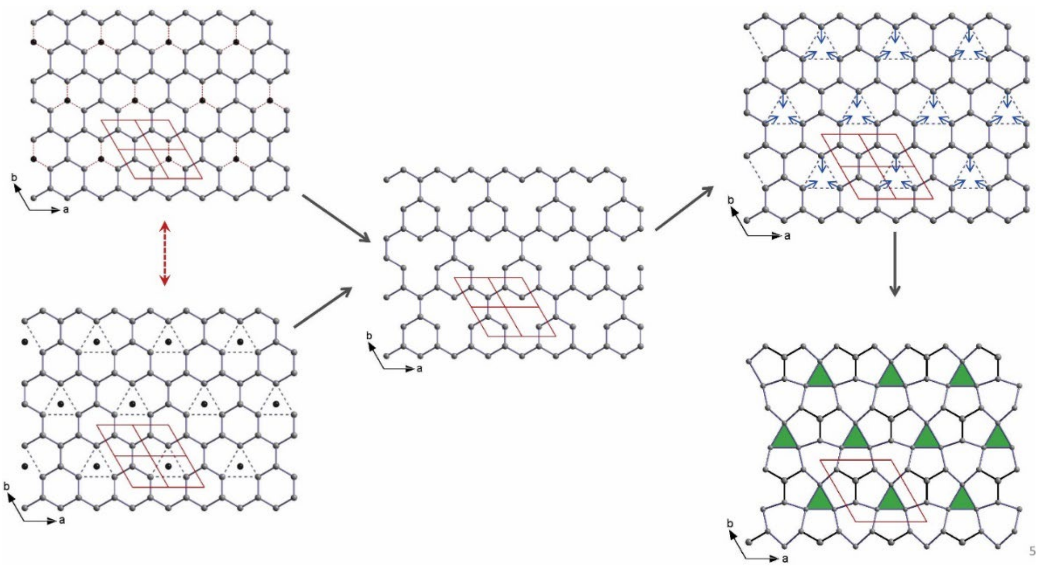

Figure S3. Atomistic modelling of Mg defect formation and ordering in the superstructure $Mg_7Pt_4Ge_4$ ($P6_3mc$) starting from the simpler Mg_2PtGe ($P6_3/mmc$).

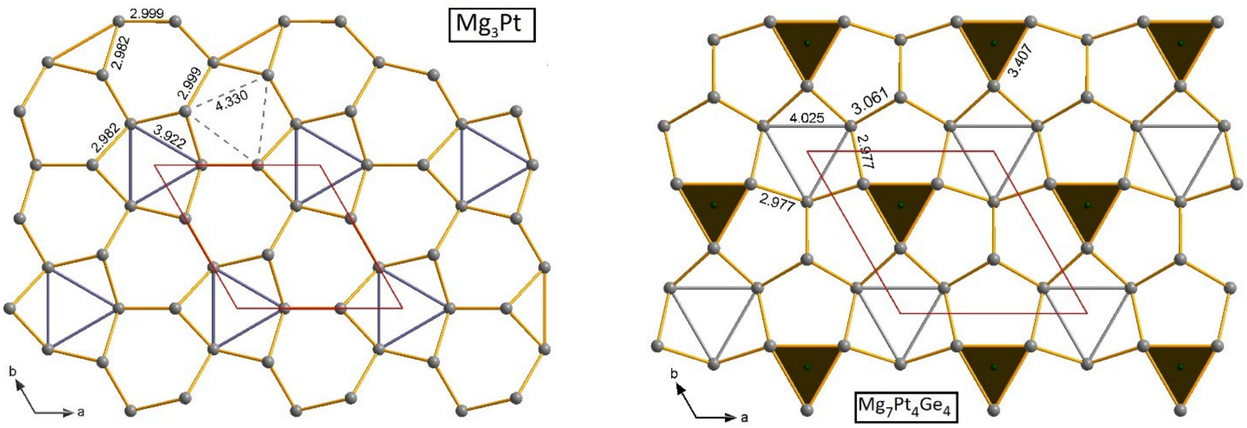

Figure S4. Comparative view of the distorted Mg honeycomb layers in the binary Mg_3Pt (left) and the defective $Mg_7Pt_4Ge_4$ (right). In Mg_3Pt, 2/3 of the hexagons are irregular due to contraction of some Mg–Mg distancesgs. In $Mg_7Pt_4Ge_4$ originates from defect positions (indicated at the centre of shaded triangular rings). Interatomic distances in Å.

Mg$_3$Pt

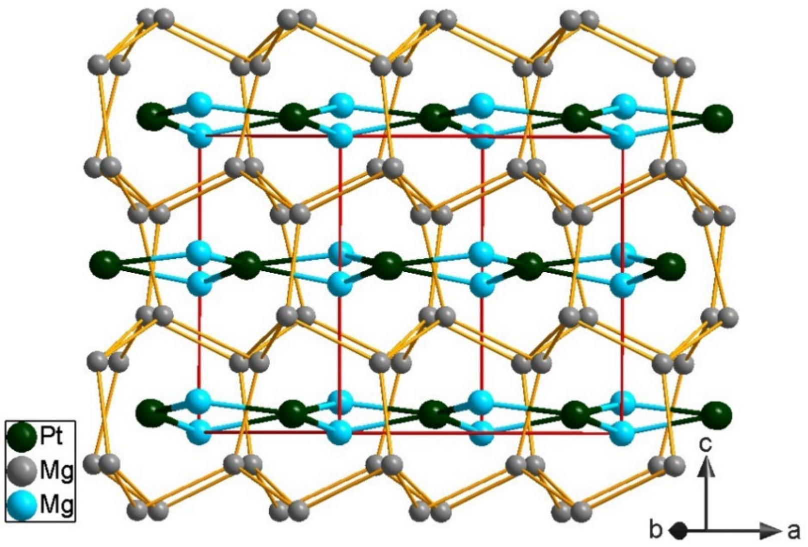

Mg$_7$Pt$_4$Ge$_4$

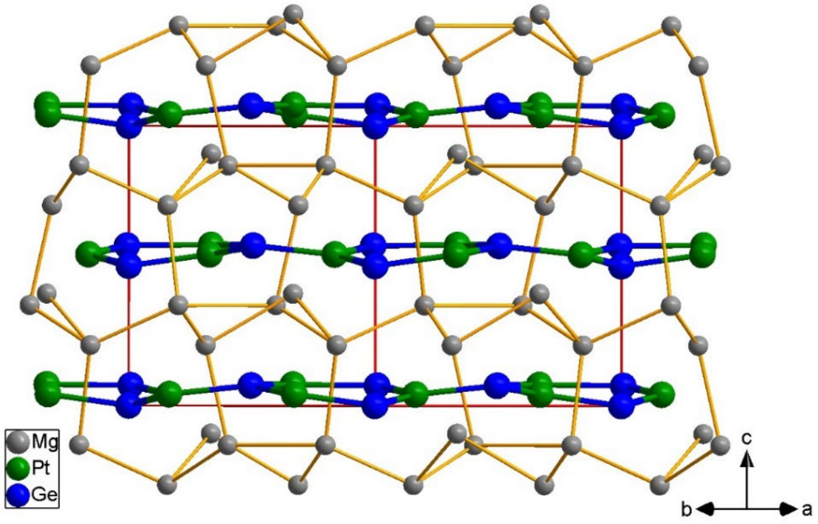

Figure S5. Similarities and differences between binary Mg$_3$Pt (top) and its ternary derivative Mg$_7$Pt$_4$Ge$_4$ (bottom). Some Mg positions (magenta) in Mg$_3$Pt are replaced by Ge (Si or), in corresponding defective phases Mg$_7$Pt$_4$Ge$_4$ (or Mg$_2$PtSi).

S3. Electronic-Structure Calculations

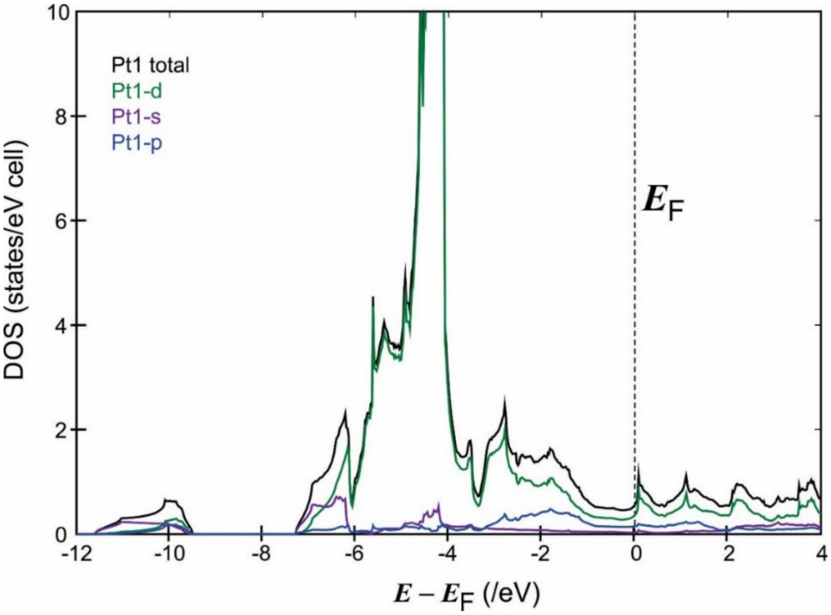

Figure S6. Projected DOS for Pt atoms, showing significant hybridization of Pt s-d-p atomic orbitals.

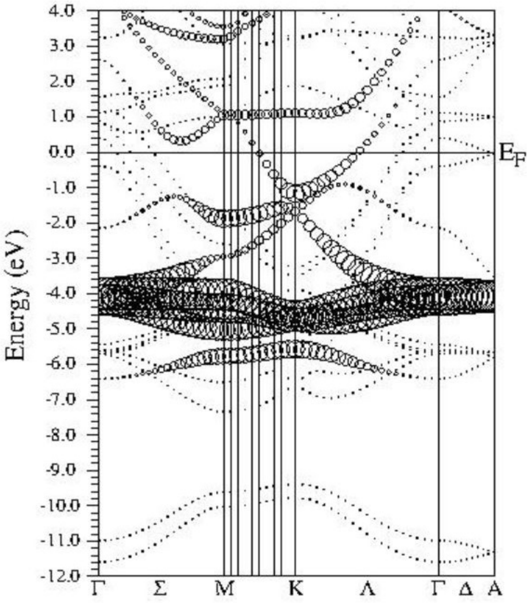

Figure S7. Fat band representation of the Pt 5d contribution to the band structure of hypothetical "Mg_2PtGe" ($P6_3/mmc$).

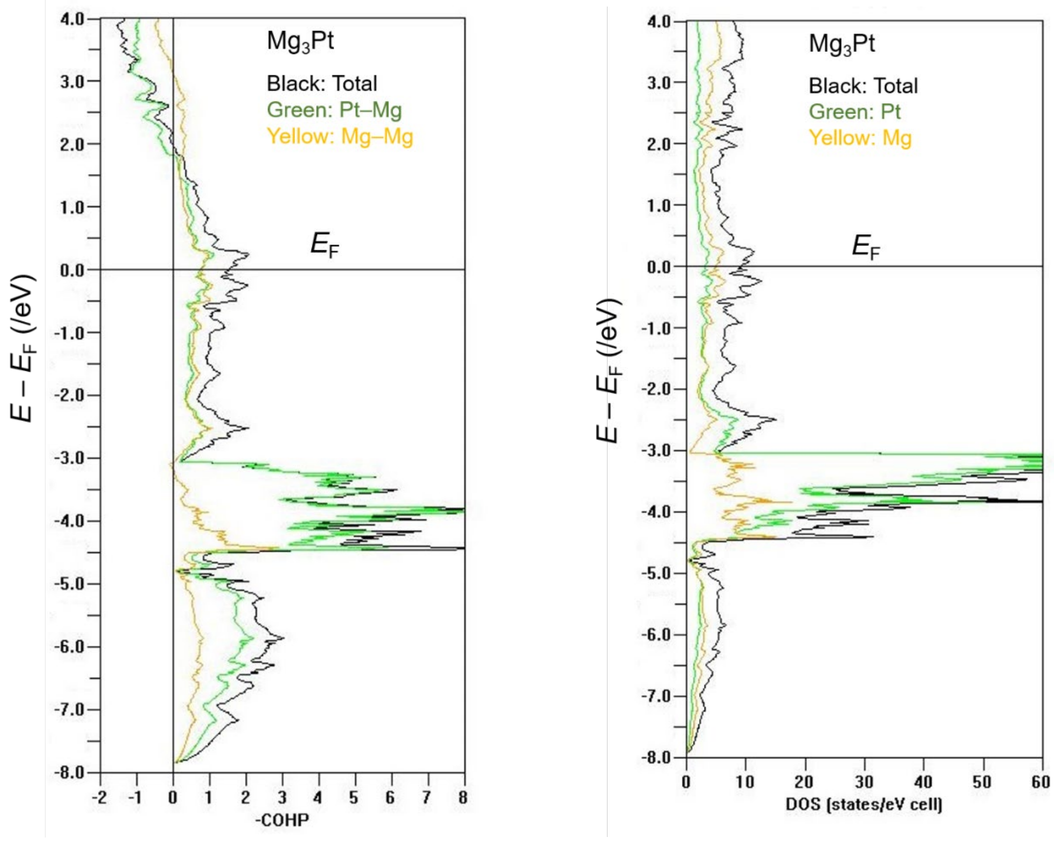

Figure S8. COHP plot of selected interactions in Mg_3Pt. All bonding levels will be occupied roughly at 2 eV above E_F, which according to IDOS corresponds to (109 ve/cell) 18,1666 ve/f.u, significantly different from Mg_2PtSi and $Mg_7Pt_4Ge_4$.

Table S4. Selected interatomic distances and ICOPH values for hypothetical defect-free "Mg_2PtGe".

**Atom pairs**		**Distances Å**	–ICOHP (/eV)	
Pt1/Ge1	3×	2.506	2.36	29 %
Pt1/Mg1	2×	2.807	1.16	↓
Pt1/Mg1	6×	2.894	0.85	31 %
Ge1/Mg1	6×	2.894	0.76	19 %
Mg1/Mg1	3×	2.851	0.72	21 %
Mg1/Mg1		2.895	0.37	

S4. Magnetic Measurements

Magnetic susceptibility measurements were carried out with a Quantum Design MPMS SQUID magnetometer. Polycrystalline sample of $Mg_7Pt_4Ge_4$ was used for the magnetic measurements. Temperature dependent data were collected for both zero field cooled (ZFC) and field cooled mode (FC) between 1.9 and 300 K, with applied field of 1 T. A typical measurement consists of initial cooling from room temperature (300 K) to 1.9 K with no applied field, and then the selected field is turned on and the ZFC data are collected on warming, followed by cooling back to 1.9 K and collect FC data on warming with the applied field still on.

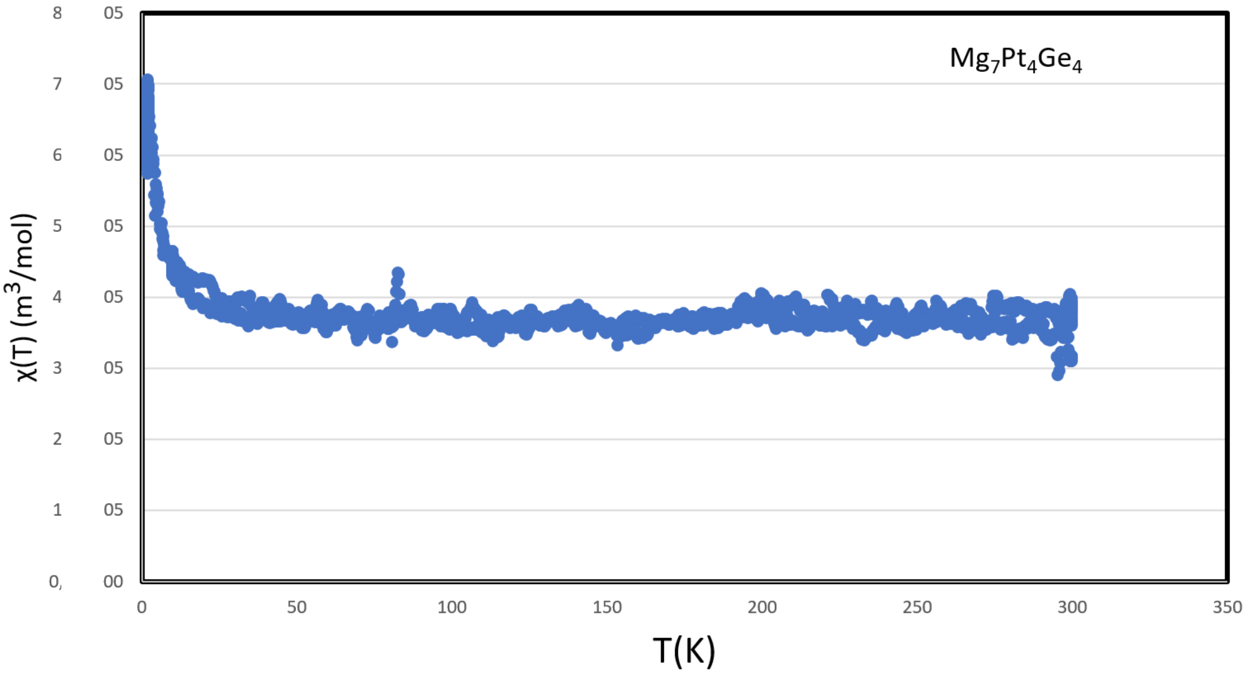

Figure S9. Temperature-dependent magnetic susceptibility χ(T) for $Mg_7Pt_4Ge_4$.
